# The Neuroses of the Genito-urinary System

**Published:** 1890-09

**Authors:** 


THE NEUROSES OF THE GENITO-URINARY SYSTEM IN THE MALE, WITH STERILITY AND IMPOTENCE. By Dr. R. Ultzmann, Professor of Genito-Urinary Diseases in the University of Vienna. Translated by Gardner W. Allen, M. D., Surgeon in the Genito-Urinary Department of Boston Dispensary. Philadelphia: 1231 Filbert Street. F. A. Davis, publisher. 1889.

This is the best work upon this subject which we have met with since the celebrated work of Acton. It gives the clearest information possible, and is most excellent for the physician of any school of medicine. Those portions of the treatment which cannot be indorsed by the consistent homœopathist can be ignored, whilst the instruction in diagnosis, hygiene, etc., should be carefully studied.

It is not a suitable book for the laity, but for the educated practitioner it must prove a great help.

It is a very small book, and can be slipped into the coat pocket, and so is available for perusal when upon a journey, waiting for a train, and the like.

